# Effects of acute high-altitude exposure on heart rate variability: a systematic review and meta-analysis

**DOI:** 10.3389/fphys.2025.1696346

**Published:** 2026-01-05

**Authors:** Hao Li, Xiangwei Chen, Cong Huang, Weiping Du

**Affiliations:** 1 School of Physical Education, Shaanxi Normal University, Xi’an, China; 2 School of Physical Education, Hong he University, Mengzi, Yunnan, China; 3 School of Physical Education, Ningxia Normal University, Guyuan, Ningxia, China

**Keywords:** high-altitude exposure, heart rate variability, autonomic nervous system, acute mountain sickness, meta-analysis

## Abstract

**Objective:**

This study aimed to systematically review and meta-analyze the effects of acute high-altitude exposure on heart rate variability (HRV), in order to elucidate the adaptive changes of the autonomic nervous system under high-altitude environments.

**Methods:**

Following the PRISMA 2020 guidelines, PubMed, Web of Science, Cochrane Library, Embase, China National Knowledge Infrastructure (CNKI), and Wanfang databases were searched from inception to June 2025. Eligible studies included healthy adults who acutely ascended to high altitude (≥2,500 m, exposure duration ≤7 days) and reported HRV-related indices, including Standard Deviation of Normal-to-Normal intervals (SDNN), Root Mean Square of Successive Differences (RMSSD), Percentage of successive NN intervals differing by more than 50 ms (pNN50), High-Frequency power (HF), and Low-Frequency power (LF). Statistical analyses were performed using Stata 17.0 and RevMan 5.4. Effect sizes were expressed as standardized mean differences (SMDs) with 95% confidence intervals (CIs). Subgroup analyses, sensitivity analyses, and publication bias assessments were also conducted.

**Results:**

A total of 15 studies involving 698 participants were included. Meta-analysis revealed that after acute high-altitude exposure, SDNN, RMSSD, pNN50, HF, and LF were all significantly (all p < 0.001) reduced compared with sea-level values, whereas the LF/HF ratio increased significantly (p < 0.001). Altitude subgroup analysis indicated that at ≥3500 m, SDNN decreased more and LF/HF increased more than at <3,500 m (p < 0.05), while no significant between-group differences were found for RMSSD, pNN50, HF, or LF (all p > 0.05). In the fitness subgroups, SDNN, RMSSD, pNN50, and HF did not differ significantly between trained individuals and healthy adults (all p > 0.05), although trained individuals exhibited a smaller reduction in LF and a more pronounced increase in LF/HF (p < 0.05). Sensitivity analyses confirmed the robustness of the results, and no obvious publication bias was detected.

**Conclusion:**

Acute high-altitude exposure markedly reduces both time- and frequency-domain HRV indices, accompanied by an increase in the LF/HF ratio, indicating an autonomic response characterized by “reduced variability, vagal withdrawal, and relative sympathetic predominance.” This response becomes more pronounced at higher elevations (≥3,500 m). Both trained and healthy adults experience vagal inhibition; however, trained show better preservation of low-frequency oscillations and stronger sympathetic regulatory capacity. Accordingly, strategies such as gradual ascent, maintaining stable breathing rhythms, and incorporating recovery-focused training before and after entering high altitude may help mitigate autonomic disturbances and facilitate early acclimatization.

**Systematic Review Registration:**

https://inplasy.com/projects/, identifier INPLASY202590004.

## 1 Introduction

The high-altitude environment, characterized by low barometric pressure, hypoxia, cold temperatures, and aridity, poses significant challenges to human physiological adaptation, particularly affecting cardiovascular, respiratory, and autonomic nervous system (ANS) functions. When individuals ascend to altitudes above 2,500 m, the body must rapidly adjust oxygen delivery and metabolic balance to cope with environmental changes (Aksel et al., 2019; Wu et al., 2014; Li et al., 2020; Bhaumik et al., 2013). During this process, the ANS undergoes substantial remodeling to regulate cardiovascular and respiratory functions. Such remodeling not only ensures cardiovascular stability but may also directly influence the risk of altitude-related illnesses, particularly acute mountain sickness (AMS) (Wu et al., 2018; Zieliński and Byś, 2020).

With increasing altitude, oxygen availability gradually decreases, and the body must adjust autonomic nervous system activity to enhance cardiovascular and respiratory function in order to maintain metabolic homeostasis. The sympathetic nervous system typically becomes more active to support oxygen supply and metabolic demands, while parasympathetic activity correspondingly decreases, leading to sympathetic dominance (Wehrwein et al., 2016; Benarroch, 2020; Herzig et al., 2024). Although such autonomic imbalance may help maintain short-term physiological stability, prolonged dysregulation compromises adaptability and can exacerbate AMS symptoms such as headache, vomiting, and insomnia (Wu et al., 2018; Kanai et al., 2001). Moreover, individuals exhibit substantial variability in their capacity to adapt to high altitude, which may be closely linked to inter-individual differences in autonomic responses. Variations in sympathetic and parasympathetic activity contribute to divergent physiological outcomes under identical high-altitude conditions (Boos et al., 2018; Roo et al., 2011). Thus, the regulatory mechanisms of the autonomic nervous system at high altitude, particularly the dynamic interplay between sympathetic and parasympathetic activity, serve as a key pathway for understanding the physiological responses to acute high-altitude exposure.

Heart rate variability (HRV) is an important physiological marker that reflects the regulatory balance of ANS and is widely used to assess the interaction between sympathetic and parasympathetic activity. HRV is primarily quantified using time- domain indices and frequency-domain indices (Tiwari et al., 2021; Roo et al., 2011). Following high-altitude exposure, HRV indices typically exhibit marked alterations, reflecting adaptive responses of the sympathetic and parasympathetic nervous systems under high altitude conditions. For example, studies have shown that acute ascent to high altitude stimulates sympathetic activation, thereby enhancing pulmonary and systemic vasoconstriction and indicating sympathetic predominance (Jin et al., 2024; Trimmel et al., 2011; Long et al., 2006b). This effect is reflected in time-domain indices: Standard Deviation of Normal-to-Normal intervals (SDNN) generally represents overall autonomic modulation, whereas Root Mean Square of Successive Differences (RMSSD) is more strongly associated with vagal activity (Trimmel et al., 2011; Long et al., 2008). Accordingly, significant reductions in SDNN and RMSSD after acute exposure suggest a decline in overall autonomic regulation, with the decrease in RMSSD particularly indicating suppressed parasympathetic activity, ultimately impairing cardiac autonomic control. These alterations may serve as early warning signals for altitude-related illnesses and underscore the complexity of physiological adaptation (Tsai et al., 2025).

In terms of frequency-domain measures, Low Frequency power (LF) primarily reflects sympathetic modulation, whereas High Frequency power (HF) is mainly indicative of parasympathetic activity (Cao et al., 2022). Under hypoxic stress, enhanced sympathetic drive is manifested as an increase in LF power, while reduced parasympathetic activity results in decreased HF power. Furthermore, the LF/HF ratio is widely employed to evaluate sympathovagal balance. Studies consistently report an elevated LF/HF ratio after high-altitude exposure, pointing to a shift toward sympathetic predominance (Huang et al., 2010). These changes highlight the profound impact of high altitude on ANS regulation, characterized by sympathetic activation and parasympathetic suppression (Jin et al., 2024; Trimmel et al., 2011). In addition, sympathetic activation is closely tied to physiological adaptation during acute exposure. Hypoxia enhances sympathetic drive, inducing pulmonary and systemic vasoconstriction—a phenomenon reflected in elevated LF power (Jin et al., 2024). Conversely, reductions in HF indicate weakened parasympathetic modulation, which has been associated with the development of AMS (Tian et al., 2008).

Taken together, HRV indices such as SDNN, RMSSD, LF, HF, and LF/HF provide valuable insights into sympathovagal interactions under hypoxic stress. Investigating their alterations is essential for understanding the adaptive capacity of the ANS in high-altitude environments.

Despite numerous studies, the findings on high-altitude exposure and HRV remain inconsistent. Some report significant alterations in both time-domain and frequency-domain indices (e.g., SDNN, RMSSD, LF/HF ratio), whereas others attribute variability to individual differences and fail to observe clear effects (Tsai et al., 2025). Such inconsistencies may arise from differences in altitude, duration of exposure, and study design. For example, some studies have observed marked HRV changes at altitudes above 2,500 m (Zhang et al., 2014), whereas others reported no significant alterations even above 3,000 m (Kiran and Medabala, 2025).

Another major limitation is that many studies were conducted in simulated high-altitude environments (e.g., normobaric or hypobaric hypoxia chambers), which do not fully reproduce the complex conditions of real altitude, such as low barometric pressure, large diurnal temperature fluctuations, and aridity. These factors may strongly influence ANS regulation (Zhang et al., 2014; Botek et al., 2015; Aebi et al., 2020). Consequently, findings from simulated settings may have limited external validity and may not adequately reflect the multifactorial stressors encountered in natural high-altitude environments.

Based on the considerations above, the study formulated the following hypotheses: acute high-altitude exposure would induce significant alterations in overall HRV, characterized by decreases in vagal-related indices (such as RMSSD and HF) and increases in sympathetic-related indices (such as LF and LF/HF). These changes would be associated with autonomic activation triggered by acute hypoxia, reflecting a pattern of sympathetic excitation and vagal withdrawal. Furthermore, variations are expected across studies conducted at different altitudes and among populations with different fitness levels.

Therefore, the present study employed a systematic review and meta-analysis to comprehensively evaluate the effects of acute real-world high-altitude exposure on HRV. Unlike previous research, this study focused exclusively on exposures above 2,500 m and integrated evidence from multiple field studies. By applying strict inclusion criteria—particularly emphasizing natural high-altitude environments and acute exposure parameters (altitude and duration)—we aimed to resolve existing inconsistencies and provide robust, generalizable conclusions. This approach offers systematic and comprehensive evidence to advance understanding of ANS and cardiovascular adaptations to acute high-altitude exposure.

## 2 Methods

### 2.1 Study design and registration

This study was conducted as a systematic review and meta-analysis, and reported in accordance with the Preferred Reporting Items for Systematic Reviews and Meta-Analyses (PRISMA) guidelines (Page et al., 2021). The analyses were performed with reference to the Cochrane Handbook for Systematic Reviews of Interventions (6th edition) (Higgins et al., 2022).

This systematic review has been registered with the International Platform of Registered Systematic Review and Meta-analysis Protocols (INPLASY) under the registration number INPLASY202590004.

### 2.2 Search strategy

Following PRISMA requirements, a systematic search of the literature related to acute high-altitude exposure was conducted. The databases searched included PubMed, Web of Science, Cochrane Library, Embase, China National Knowledge Infrastructure (CNKI), Wanfang, from inception to June 2025. The search focused on comparative studies of HRV at baseline (sea level) and after ascent to high altitude. Eligible participants were required to be lowland adults with HRV data available both before and within 7 days after acute high-altitude exposure. Both MeSH terms and free-text terms were used in the search strategy (Supplementary Table S1).

For example, the PubMed search strategy was: (Altitude[MeSH] OR Hypoxia[MeSH] OR High altitude OR Plateau OR Hypobaric hypoxia OR Acute mountain sickness) AND (Heart Rate Variability[MeSH] OR HRV OR Heart rate variability OR Cardiac autonomic function OR Autonomic nervous system) AND (Acute[All Fields] OR Short-term[All Fields] OR Immediate[All Fields]).

### 2.3 Inclusion and exclusion criteria

Inclusion criteria: (1) Study type: Clinical studies, including randomized controlled trials (RCTs), non-randomized controlled trials, prospective or retrospective cohort studies. (2) Participants: Healthy adults or volunteers without severe underlying diseases. (3) Exposure: Acute ascent to high altitude, altitude ≥2,500 m, exposure duration ≤7 days. (4) Control: Sea-level baseline (≤600 m) or pre-exposure values from the same participants. (5) Outcomes: At least one HRV parameter reported, including but not limited to SDNN, RMSSD, pNN50(Percentage of successive NN intervals differing by more than 50 ms), LF, HF, LF/HF.

Exclusion criteria: (1) Reviews, case reports, conference abstracts, and animal studies. (2) Studies involving participants with major comorbidities (e.g., cardiovascular disease, neurological disorders, severe infections) without separate data for healthy populations. (3) Studies lacking extractable quantitative HRV data (mean ± SD or median ± IQR) and where authors could not be contacted for additional data. (4) Long-term high-altitude residents or chronic exposure studies not meeting the definition of “acute exposure.”

### 2.4 Data extraction

Data extraction was conducted independently by two researchers (HL and XwC. The extracted information included: (1) Basic study information: first author, year of publication, country or region, sample size, participant characteristics (sex and age), and study design. (2) Exposure characteristics: mode of ascent, altitude reached, and duration of exposure. (3) Outcome measures: mean and standard deviation (or convertible data) of HRV parameters reported in each study. When discrepancies arose between the two researchers, a third reviewer (WpD or CH) verified the extracted information and resolved disagreements to ensure accuracy and consistency. (4) To enhance the comparability of HRV data across studies, measurement conditions were standardized with reference to the Task Force recommendations. Key aspects included the mode of ascent (e.g., vehicle, cable car, or air transport), type and duration of ECG recording (e.g., 5-min ECG), body position during measurement (sitting or supine), pre-measurement resting period (typically 5–40 min), control of confounding factors (e.g., exclusion of medication use, caffeine intake, smoking, or recent exercise), time of assessment (daytime recordings such as 08:00–21:00, or nighttime monitoring), and participants’ Fitness level (e.g., healthy adults or trained). General characteristics of the included studies are presented in Table 1.

**TABLE 1:** Basic characteristics of included studies.

Author and years	Country/ Region	Age	Genders	Design	Population	Sample size(n)		Exposure duration	Exposure site	HRV outcomes	HRV detection conditions
						Exposure group	Control group				
Trimmel et al. (2011)	Austria	41.2 ± 19.9	Male/female	Control	Resident	45	45	Day 1	Dachstein (2700 m)	a,b,c,d,e,f	Arrival: Cable car; Protocol: 5-min ECG; Position: Seated; Resting: 40 min; Confounders: No meds; Circadian: 08:00–21:00; Fitness level: Healthy adults
Tian et al. (2008)	China	18.2 ± 0.8	Male	Control	Youth	94	86	Day 2–4	Xizang (3700 m)	a,b,c,d,e,f	Arrival: Airplane; Protocol: 5-min ECG; Position: Seated; Resting: 15 min; Confounders: No exercise/stimulants; Circadian: NR; Fitness level: Trained
Long et al. (2006a)	China	19.0 ± 0.96	Male	Control	Army soldier	86	86	Day 2–4	Xizang (3675 m)	a,b,c,d,e,f	Arrival: Airplane; Protocol: 5-min ECG; Position: Supine; Resting: ≥15 min; Confounders: No exercise/stimulants; Circadian: NR; Fitness level: Trained
Li et al. (2020)	China	18–35	Male	Control	Resident	106	106	Day 1	Qinghai (4000 m)	a,c	Arrival: Train + Bus; Protocol: 24-h ECG; Position: Ambulatory; Resting: NR; Confounders: No diseases/stimulants; Circadian: 24 h; Fitness level:Healthy adults
Chen et al. (2008)	China	39 ± 12	Male/female	Control	Resident	27	27	Day 2	Taiwan (3180 m)	a,d,e,f	Arrival: Car; Protocol: 10-min ECG; Position: Supine;Resting: 10 min; Confounders: No caffeine/tea; Circadian: 21: 00; Fitness level: Healthy adults
Boos et al. (2018)	Switzerland	31.4 ± 8.1	Male	Control	Army soldier	16	16	Day 2–3	Berne (2840 m)	a,b,c,d,e,f	Arrival: Cable car; Protocol: 2:00–3:00am; Position: Supine; Resting: Natural sleep; Confounders: No diet/caffeine/smoking; Circadian: Night; Fitness level: Trained
Hamm et al. (2018)	Germany	34.5 ± 2.5	Male/female	Control	Resident	8	8	Day 1	Zugspitze (2650 m)	a,b,d,e,f	Arrival: Train; Protocol: 30-min ECG; Position: Supine; Resting: 10–15 min; Confounders: No meds/caffeine/smoking; Circadian: 21:00pm; Fitness level: Healthy adults
Shi et al. (2020)	China	18–35	Male	Control	Army soldier	91	40	Day 1	Qinghai (4000 m)	a,b,c	Arrival: Rapid ascent; protocol: 24-h ECG; Position: Ambulatory; resting: Ambulatory; confounders: No exercise/stimulants; circadian: 24h; fitness level: Trained
Sareban et al. (2020)	Austria	31 ± 7	Male	Control	Athlete	19	19	Day 1–2	Switzerland (3450 m)	a,b,c,f	Arrival: Train; Protocol: 5-min ECG; Position: Supine;Resting: 20 min; Confounders: No meds/smoking/alcohol/caffeine; Circadian: Multiple sessions; Fitness level: Trained
Sareban et al. (2020)	Austria	38 ± 9	Male	Control	Resident	19	19	Day 1–2	Switzerland (3450 m)	a,b,c,f	Arrival: Train; Protocol: 5-min ECG; Position: Supine;Resting: 20 min; Confounders: No meds/smoking/alcohol/caffeine; Circadian: Multiple sessions; Fitness level: Healthy adults
Long et al. (2008)	China	18.3 ± 0.84	Male	Control	Army soldier	86	86	Day 2–4	Xizang (3675 m)	a,b,c,d,e,f	Arrival: Airplane; Protocol: 5-min ECG; Position: Supine;Resting: ≥15 min; Confounders: No exercise/stimulants; Circadian: NR; Fitness level: Trained
Huang et al. (2010)	Nepal	49.3 ± 2.3	Male/female	Control	Office workers	32	32	Day 5	Nepal (3440 m)	d,e,f	Arrival: Airplane; Protocol: 5-min ECG; Position: Supine; Resting: >2 h; Confounders: No diseases/stimulants; Circadian: Daytime; Physicalactivity level: Healthy adults
Long et al. (2006b)	China	17–20	Male	Control	Army soldier	86	86	Day 2–4	Xizang (3675 m)	a,b,c,d,e,f	Arrival: Airplane; Protocol: 5-min ECG; Position: Supine;Resting: ≥15 min; Confounders: No exercise/stimulants; Circadian: Daytime; Fitness level: Trained
Bhaumik et al. (2013)	Ladakh	24.83 ± 2.93	Male	Control	Army soldier	6	6	Day2, 5	Leh (3500 m)	d,e,f	Arrival: Airplane; Protocol: 5-min ECG; Position: Supine;Resting: After stable ECG baseline; Confounders: No exercise/stimulants; Circadian: Daytime; Fitnesslevel: Trained
Herzig et al. (2024)	Switzerland	60.7 ± 13.6	Male /Female	Control	Resident	24	24	Day 1	Mount Säntis (2500 m)	b,d,e,f	Arrival: Cable car; Protocol: Continuous ECG (5-min HRV segments); Position: Supine; Resting: Supine; Confounders: No exercise/stimulants; Circadian: Day and night; Fitness level: Healthy adults
Kanai et al. (2001)	Japan	35 ± 5	Male/female	Control	Office workers	12	12	Day 1	NR (2700 m)	d,e,f	Arrival: Car; Protocol: 10-min ECG; Position: Supine;Resting: >2 h Confounders: No diseases/stimulants; Circadian: Daytime; Fitness level: Healthy adults

1. a = SDNN (standard deviation of normal-to-normal intervals); b = RMSSD (root mean square of successive differences between adjacent NN intervals); c = pNN50 (percentage of adjacent NN intervals differing by more than 50 ms); d = LF (low-frequency power); e = HF (high-frequency power); f = LF/HF (ratio of low-frequency to high-frequency power).

2. NR indicates “Not Reported”; Arrival indicates the mode of transportation to the high-altitude area; Protocol indicates the recording duration and analytical signal of heart rate variability; Position indicates the body position during measurement; Resting indicates the rest period before measurement initiation; Confounders indicates the confounding factors controlled for in the study; Circadian indicates the time of day during which measurements were conducted; Fitness level indicates whether participants were trained or healthy adults.

### 2.5 Outcome measures

The primary outcomes of this study were HRV parameters, encompassing both time-domain and frequency-domain indices. Time-domain measures included SDNN, RMSSD, and pNN50: SDNN reflects overall heart rate variability, whereas RMSSD and pNN50 are commonly used to assess cardiac vagal activity. Frequency-domain indices included HF, LF, and LF/HF. HF represents vagally mediated respiratory modulation, LF is influenced by multiple autonomic mechanisms, and LF/HF is often used to characterize the directional shifts in sympathetic–vagal regulation (The primary outcomes of this study were HRV parameters, encompassing both time-domain and frequency-domain indices. Time-domain measures included SDNN, RMSSD, and pNN50: SDNN reflects overall heart rate variability, whereas RMSSD and pNN50 are commonly used to assess cardiac vagal activity. Frequency-domain indices included HF, LF, and LF/HF. HF represents vagally mediated respiratory modulation, LF is influenced by multiple autonomic mechanisms, and LF/HF is often used to characterize the directional shifts in sympathetic–vagal regulation (Task Force of the European Society of Cardiology and the North American Society of Pacing and Electrophysiology, 1996). These parameters are widely applied in studies of acute high-altitude exposure and serve as essential quantitative indicators for evaluating autonomic responses and short-term hypoxic stress. Therefore, they were selected as the core outcome variables of this study.

### 2.6 Quality assessment

To comprehensively evaluate the quality and reliability of the included studies, the ROBINS-I (Risk of Bias in Non-randomized Studies of Interventions) tool was applied to all non-randomized intervention studies (non-RCTs) (Sterne et al., 2016). This tool is designed to assess potential sources of bias before, during, and after the intervention in observational research. It covers seven domains: (1) bias due to confounding; (2) bias in the selection of participants; (3) bias in the classification of interventions; (4) bias due to deviations from intended interventions; (5) bias due to missing data; (6) bias in the measurement of outcomes; and (7) bias in the selection of the reported result. Each domain is rated as having low, moderate, serious, or critical risk of bias, and an overall judgment is derived accordingly. Low risk indicates adequate study design and reporting with minimal likelihood of bias; moderate risk suggests some limitations that are unlikely to materially affect the conclusions; serious risk reflects substantial methodological concerns or insufficient control of confounding, reducing the credibility of the findings; and critical risk denotes a level of bias severe enough to undermine the interpretability of the results. Detailed assessments are presented in Table 2.

**TABLE 2:** Risk of bias assessment for included studies (based on the ROBINS-I tool).

Study	Pre - intervention		At intervention	Post - intervention				Overall bias
Author and years	Bias due to confounding	Bias due to selection of participants	Bias in classification of interventions	Bias due to deviations from intended interventions	Bias due to missing data	Bias in measurement of outcomes	Bias in selection of the report result	Low/moderate/ serious/critical
Trimmel et al. (2011)	Moderate	Low	Low	Low	Low	Low	Low	Moderate
Tian et al. (2008)	Low	Low	Low	Low	Low	Low	Low	Low
Long et al. (2006a)	Low	Low	Low	Low	Low	Low	Low	Low
Li et al. (2020)	Moderate	Low	Low	Low	Low	Low	Low	Moderate
Chen et al. (2008)	Moderate	Low	Low	Low	Low	Low	Low	Moderate
Boos et al. (2018)	Low	Low	Low	Low	Low	Low	Low	Low
Hamm et al. (2018)	Moderate	Low	Low	Low	Low	Low	Low	Moderate
Shi et al. (2020)	Moderate	Moderate	Low	Low	Low	Low	Low	Moderate
Sareban et al. (2020)	Moderate	Low	Low	Low	Low	Low	Low	Moderate
Long et al. (2008)	Low	Low	Low	Low	Low	Low	Low	Low
Huang et al. (2010)	Moderate	Moderate	Moderate	Low	Low	Low	Low	Moderate
Long et al. (2006b)	Low	Low	Low	Low	Low	Moderate	Low	Moderate
Bhaumik et al. (2013)	Moderate	Moderate	Low	Low	Low	Low	Low	Moderate
Herzig et al. (2024)	Low	Moderate	Low	Low	Low	Low	Low	Moderate
Kanai et al. (2001)	Moderate	Moderate	Moderate	Low	Low	Low	Low	Moderate

Green cells indicate low risk of bias, and yellow cells indicate moderate risk of bias.

### 2.7 Statistical analysis

Statistical analyses were performed using Stata 17.0 (StataCorp LLC, College Station, TX, USA) and RevMan 5.4 (The Cochrane Collaboration, London, UK). Meta-analyses were conducted using the metan and metareg functions in Stata. The primary effect size was expressed as the standardized mean difference (SMD) with corresponding 95% confidence intervals (CIs). Heterogeneity was assessed using the Q test and the I^2^ statistic. A fixed-effects model was applied when I^2^ < 50% and p > 0.10; otherwise, the DerSimonian–Laird random-effects model was used.

To evaluate the robustness of the results, sensitivity analysis was performed using the metainf function with a leave-one-out approach, examining the influence of each individual study on the pooled effect size. Subgroup analyses were conducted for altitude and fitness levels. Publication bias was assessed using Begg’s test and Egger’s regression test (Begg and Mazumdar, 1994; Egger et al., 1997), supplemented by visual inspection of funnel plot symmetry. A p-value <0.05 was considered indicative of potential publication bias.

## 3 Results

### 3.1 Literature search results

**FIGURE 1:**
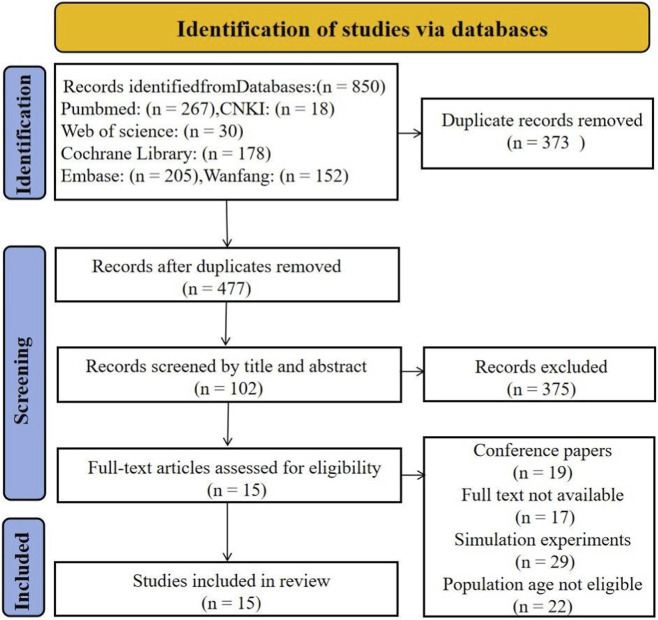
Flowchart of literature screening.

The literature screening process is illustrated in Figure 1. A total of 850 studies were identified through the predefined search strategy. After removing 373 duplicates and excluding 375 irrelevant articles based on titles and abstracts, 102 full-text articles were reviewed. Following application of the inclusion and exclusion criteria, 87 articles were excluded. Ultimately, 15 studies were included in the analysis.

### 3.2 Study characteristics

A total of 15 studies were included. Among these, 8 studies involved exposure at altitudes between 2,500 and 3,500 m, while 8 studies involved exposure above 3,500 m, comprising 698 participants overall. Notably, in the study by Sareban et al. (2020), different populations (endurance athletes vs. untrained controls) were reported separately, and thus the data were included twice. Detailed study characteristics are presented in Table 1.

### 3.3 Quality assessment

The methodological quality of the included studies was systematically evaluated using the ROBINS-I tool for assessing risk of bias in non-randomized intervention studies. As summarized in Table 2, most studies demonstrated a low risk of bias across the assessed domains, with only a few studies exhibiting moderate risk related to confounding or participant selection. Overall, the included studies were judged to have a low-to-moderate risk of bias, indicating generally high methodological quality and good credibility of the findings.

### 3.4 HRV outcomes

To systematically present the changes in heart rate variability (HRV) following acute high-altitude exposure, time-domain and frequency-domain indices, as well as altitude and fitness subgroups, were analyzed separately.

#### 3.4.1 SDNN

**FIGURE 2:**
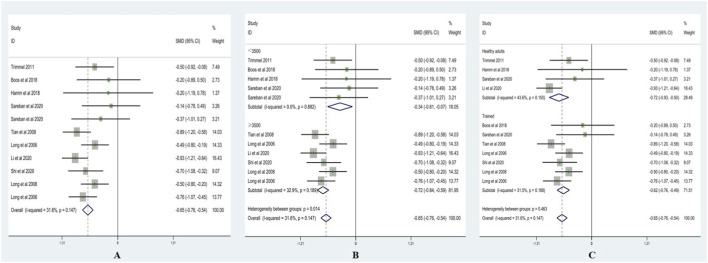
Forest plots of SDNN (**(A)** = fixed-effects model; **(B)** = altitude subgroup analysis; **(C)** = fitness subgroup analysis).

Heterogeneity testing for SDNN showed I^2^ = 31.6% (<50%) with a non-significant Q test (p > 0.10); therefore, a fixed-effects model was applied. As shown in Figure 2A, SDNN was significantly lower after acute ascent compared with sea level, with SMD = −0.65 (95% CI: −0.76, −0.54), z = 11.09, p < 0.001.

Altitude subgroup analysis (Figure 2B) indicated a significant difference in SDNN between altitude categories (p < 0.05). At <3,500 m, SMD = −0.34 (95% CI: −0.61, −0.07), z = 2.50, p < 0.05 (I^2^ = 0%, p = 0.88). At ≥3,500 m, SMD = −0.72 (95% CI: −0.85, −0.59), z = 11.08, p < 0.001 (I^2^ = 32.9%, p = 0.19). These findings suggest that altitude significantly modifies the effect of acute high-altitude exposure on SDNN.

Fitness subgroup analysis (Figure 2C) showed no significant differences between fitness groups (p > 0.05). Among healthy adults, SMD = −0.72 (95% CI: −0.93, −0.50), z = 6.54, p < 0.001 (I^2^ = 43.6%, p = 0.150). Among trained individuals, SMD = −0.62 (95% CI: −0.76, −0.49), z = 8.99, p < 0.001 (I^2^ = 31.5%, p = 0.188). This indicates that fitness level did not significantly moderate the effect of acute high-altitude exposure on SDNN.

#### 3.4.2 RMSSD

**FIGURE 3:**
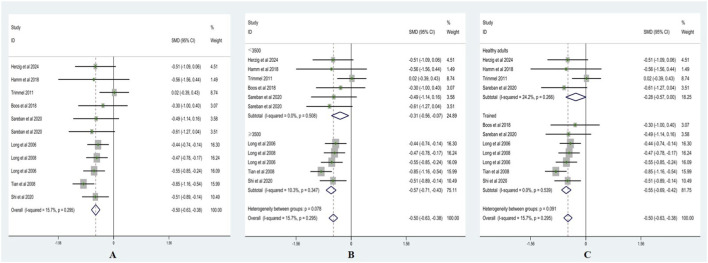
Forest plots of RMSSD (**(A)** = fixed-effects model; **(B)** = altitude subgroup analysis; **(C)** = fitness subgroup analysis).

Heterogeneity testing for RMSSD showed I^2^ = 15.7% (<50%) with a non-significant Q test (p > 0.10); therefore, a fixed-effects model was applied. As presented in Figure 3A, RMSSD was significantly lower after acute ascent compared with sea level, with SMD = −0.50 (95% CI: −0.63, −0.38), z = 8.09, p < 0.001.

Altitude subgroup analysis (Figure 3B) indicated no significant differences in RMSSD between altitude categories (p > 0.05). At <3,500 m, SMD = −0.31 (95% CI: −0.56, −0.07), z = 2.25, p < 0.05 (I^2^ = 0%, p = 0.51). At ≥3,500 m, SMD = −0.57 (95% CI: −0.71, −0.43), z = 7.89, p < 0.001 (I^2^ = 10.03%, p = 0.35). These results suggest that altitude did not significantly moderate the effect of acute high-altitude exposure on RMSSD.

Fitness subgroup analysis (Figure 3C) also showed no significant differences between fitness groups (p > 0.05). Among healthy adults, SMD = −0.28 (95% CI: −0.57, 0.00), z = 1.93, p = 0.054 (I^2^ = 24.2%, p = 0.266). Among trained, SMD = −0.55 (95% CI: −0.69, −0.42), z = 8.04, p < 0.001 (I^2^ = 0%, p = 0.539). These findings indicate that fitness level did not significantly moderate the effect of acute high-altitude exposure on RMSSD.

#### 3.4.3 pNN50

**FIGURE 4:**
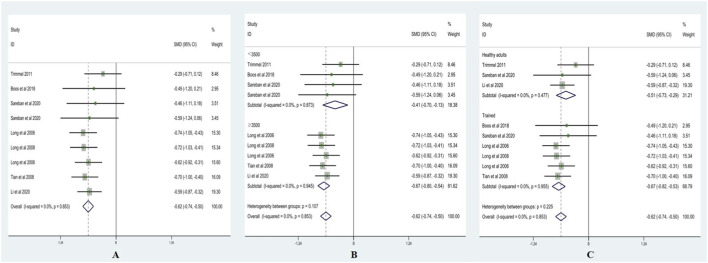
Forest plots of pNN50 (**(A)** = fixed-effects model; **(B)** = altitude subgroup analysis; **(C)** = fitness subgroup analysis).

Heterogeneity testing for pNN50 showed I^2^ = 0% (<50%) with a non-significant Q test (p > 0.10); therefore, a fixed-effects model was applied. As shown in Figure 4A, pNN50 was significantly lower after acute ascent compared with sea level, with SMD = −0.62 (95% CI: −0.74, −0.50), z = 10.09, p < 0.001.

Altitude subgroup analysis (Figure 4B) indicated no significant differences in pNN50 between altitude categories (p > 0.05). At <3,500 m, SMD = −0.41 (95% CI: −0.70, −0.13), z = 2.87, p < 0.01 (I^2^ = 0%, p = 0.87). At ≥3,500 m, SMD = −0.67 (95% CI: −0.80, −0.54), z = 9.81, p < 0.001 (I^2^ = 0%, p = 0.95). These findings suggest that altitude did not significantly moderate the effect of acute high-altitude exposure on pNN50.

Fitness subgroup analysis (Figure 4C) also showed no significant differences between fitness groups (p > 0.05). Among healthy adults, SMD = −0.51 (95% CI: −0.73, −0.29), z = 4.63, p < 0.001 (I^2^ = 0%, p = 0.477). Among trained, SMD = −0.67 (95% CI: −0.82, −0.53), z = 9.05, p < 0.001 (I^2^ = 0%, p = 0.955). These results indicate that fitness level did not significantly moderate the effect of acute high-altitude exposure on pNN50.

#### 3.4.4 HF

**FIGURE 5:**
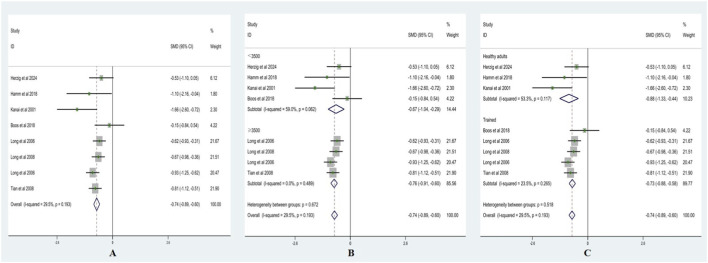
Forest plots of HF (**(A)** = fixed-effects model; **(B)** = altitude subgroup analysis; **(C)** = fitness subgroup analysis).

Heterogeneity testing for HF showed I^2^ = 29.5% (<50%) with a non-significant Q test (p > 0.10); therefore, a fixed-effects model was applied. As presented in Figure 5A, HF was significantly lower after acute ascent compared with sea level, with SMD = −0.74 (95% CI: −0.89, −0.60), z = 10.24, p < 0.001.

Altitude subgroup analysis (Figure 5B) indicated no significant differences in HF between altitude categories (p > 0.05). At <3,500 m, SMD = −0.67 (95% CI: −1.04, −0.29), z = 3.50, p < 0.001 (I^2^ = 59%, p = 0.06). At ≥3,500 m, SMD = −0.76 (95% CI: −0.91, −0.60), z = 9.81, p < 0.001 (I^2^ = 0%, p = 0.49). These findings suggest that altitude did not significantly moderate the effect of acute high-altitude exposure on HF.

Fitness subgroup analysis (Figure 5C) also showed no significant differences between fitness groups (p > 0.05). Among healthy adults, SMD = −0.88 (95% CI: −1.33, −0.44), z = 3.89, p < 0.001 (I^2^ = 53.3%, p = 0.117). Among trained, SMD = −0.73 (95% CI: −0.88, −0.58), z = 9.49, p < 0.001 (I^2^ = 23.5%, p = 0.265). These results indicate that fitness level did not significantly moderate the effect of acute high-altitude exposure on HF.

#### 3.4.5 LF

**FIGURE 6:**
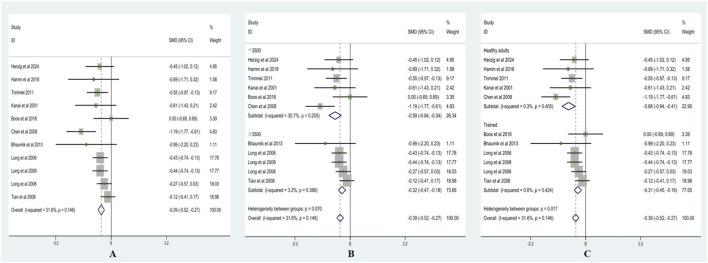
Forest plots of LF (**(A)** = fixed-effects model; B = altitude subgroup analysis; **(C)** = fitness subgroup analysis).

Heterogeneity testing for LF showed I^2^ = 31.6% (<50%) with a non-significant Q test (p > 0.10); therefore, a fixed-effects model was applied. As shown in Figure 6A, LF was significantly lower after acute ascent compared with sea level, with SMD = −0.39 (95% CI: −0.52, −0.27), z = 6.06, p < 0.001.

Altitude subgroup analysis (Figure 6B) indicated no significant differences in LF between altitude categories (p > 0.05). At <3,500 m, SMD = −0.59 (95% CI: −0.84, −0.34), z = 4.66, p < 0.001 (I^2^ = 30.7%, p = 0.21). At ≥3,500 m, SMD = −0.32 (95% CI: −0.47, −0.18), z = 4.27, p < 0.001 (I^2^ = 3.2%, p = 0.39). These findings suggest that altitude did not significantly moderate the effect of acute high-altitude exposure on LF.

Fitness subgroup analysis (Figure 6C) showed significant differences between fitness groups (p < 0.05). Among healthy adults, SMD = −0.68 (95% CI: −0.94, −0.41), z = 4.99, p < 0.001 (I^2^ = 0.3%, p = 0.405). Among trained, SMD = −0.31 (95% CI: −0.45, −0.16), z = 4.17, p < 0.001 (I^2^ = 0%, p = 0.424). These results indicate that fitness level significantly moderated the effect of acute high-altitude exposure on LF.

#### 3.4.6 LF/HF ratio

**FIGURE 7:**
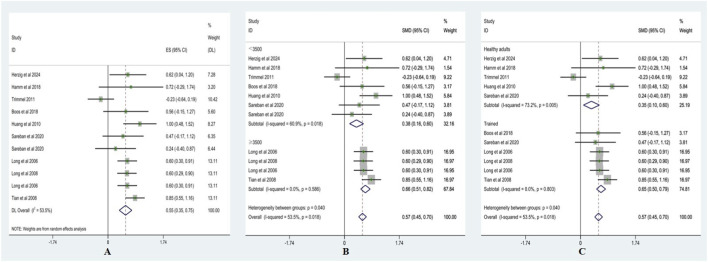
Forest plots of LF/HF (**(A)** = random-effects model; **(B)** = altitude subgroup analysis; **(C)** = fitness subgroup analysis).

Heterogeneity testing for LF/HF showed I^2^ = 53.5% (>50%) with a significant Q test (p < 0.10); therefore, a random-effects model was applied. As presented in Figure 7A, LF/HF was significantly higher after acute ascent compared with sea level, with ES = 0.55 (95% CI: 0.35, 0.76), t = 5.18, p < 0.001.

Altitude subgroup analysis (Figure 7B) revealed significant differences between altitude categories (p < 0.05). At <3,500 m, SMD = 0.38 (95% CI: 0.16, 0.60), z = 3.37, p < 0.01 (I^2^ = 60.9%, p = 0.02). At ≥3,500 m, SMD = 0.66 (95% CI: 0.51, 0.82), z = 8.51, p < 0.001 (I^2^ = 0%, p = 0.59). These findings suggest that altitude significantly moderated the effect of acute high-altitude exposure on LF/HF.

Fitness subgroup analysis (Figure 7C) also showed significant differences between fitness groups (p < 0.05). Among healthy adults, SMD = 0.35 (95% CI: 0.10, 0.60), z = 2.70, p = 0.007 (I^2^ = 73.2%, p = 0.005). Among trained, SMD = 0.65 (95% CI: 0.50, 0.79), z = 8.75, p < 0.001 (I^2^ = 0%, p = 0.803). These results indicate that fitness level significantly moderated the effect of acute high-altitude exposure on LF/HF.

**FIGURE 8:**
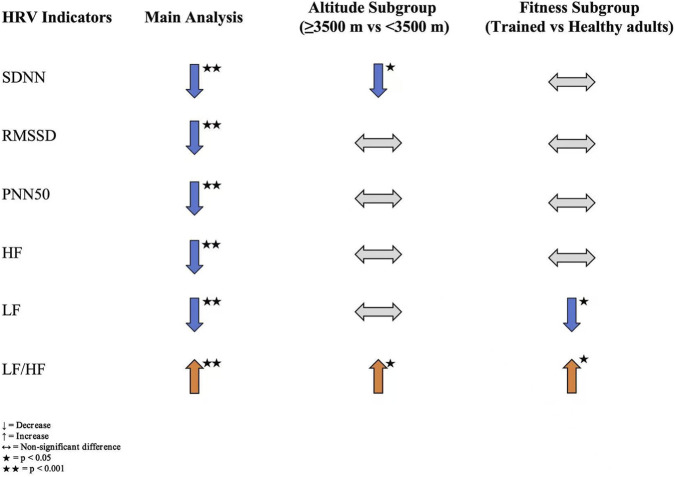
Summary of HRV changes after acute high-altitude exposure.

Taken together, acute high-altitude exposure produced a consistent pattern of changes across both time- and frequency-domain HRV indices: SDNN, RMSSD, pNN50, HF, and LF showed overall reductions, whereas LF/HF increased, reflecting an autonomic profile characterized by reduced overall variability, vagal withdrawal, and relative sympathetic predominance. Altitude primarily influenced overall variability (SDNN) and the sympathovagal balance (LF/HF), while its effects on vagal-related indices (RMSSD, pNN50, HF) were comparatively limited. Fitness level mainly affected the expression of low-frequency oscillations and sympathetic output; trained individuals exhibited a smaller reduction in LF and a greater increase in LF/HF, suggesting a distinct autonomic response pattern under acute hypoxia compared with healthy adults. Overall, the findings indicate marked autonomic disturbances following acute high-altitude exposure, with characteristic variations according to altitude and fitness background (Figure 8).

### 3.5 Sensitivity analysis

**FIGURE 9:**
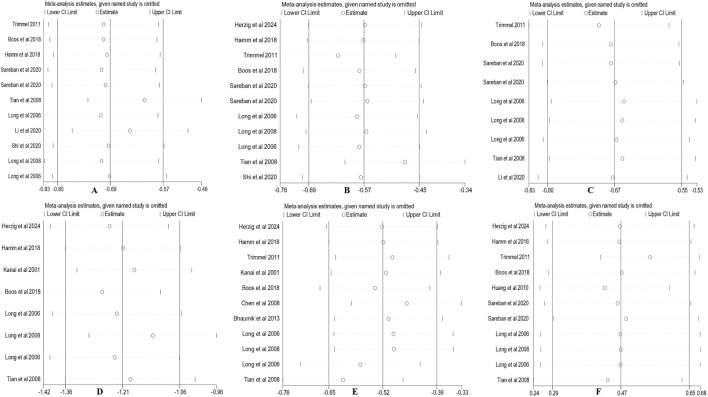
Leave-one-out sensitivity analysis plot. (Note: **(A)** SDNN; **(B)** RMSSD; **(C)** pNN50; **(D)** HF; **(E)** LF; **(F)** LF/HF).

To evaluate the robustness of the pooled effect sizes, leave-one-out sensitivity analyses were performed for all six HRV indices. The analyses were conducted under the same model specifications used in the primary meta-analyses, with fixed-effects models applied for SDNN, RMSSD, pNN50, HF, and LF, and a random-effects model applied for LF/HF. The results showed that removal of any single study did not alter the direction of the pooled effect size, and the recalculated estimates and their 95% confidence intervals largely overlapped with the overall pooled values. No individual study exerted a substantial influence on the summary effect (Figure 9). Collectively, the sensitivity analyses indicate that the findings of this meta-analysis are robust.

### 3.6 Publication bias assessment

**FIGURE 10:**
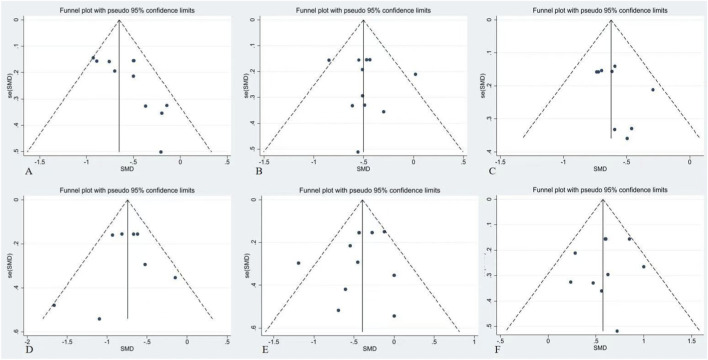
Funnel plots for publication bias assessment. (Note: **(A)** = SDNN; **(B)** = RMSSD; **(C)** = pNN50; **(D)** = HF; **(E)** = LF; **(F)** = LF/HF).

To evaluate potential publication bias among the included studies, funnel plots were generated for each HRV outcome, and Begg’s test and Egger’s regression test were performed (Figure 10). Visual inspection showed that the funnel plots for SDNN, RMSSD, pNN50, HF, LF, and LF/HF were generally symmetrical, with no clear indication of small-study effects or systematic bias. Except for SDNN, for which the slope term in Egger’s regression reached statistical significance, the p values of both Begg’s and Egger’s tests for the remaining indices were all >0.05. Overall, the primary effect estimates demonstrated reasonable stability and appeared to reflect the general trend of HRV changes following acute high-altitude exposure.

It should be noted, however, that the number of included studies for each outcome was fewer than 20, which limits the statistical power of conventional publication bias tests. Considering the funnel plot patterns, statistical test results, and the limited number of studies, the current evidence does not suggest obvious publication bias, although the possibility of underlying bias cannot be fully excluded.

## 4 Discussion

### 4.1 Effects of acute high-altitude exposure on HRV

Through systematic review and meta-analysis, this study found that acute high-altitude exposure significantly reduced the HRV indices SDNN, RMSSD, pNN50, LF, and HF, while LF/HF increased significantly. Specifically, SDNN, RMSSD, pNN50, LF, and HF were all markedly lower after acute ascent compared with sea level, whereas LF/HF was substantially higher, with all differences reaching statistical significance (p < 0.001). This pattern reflects a typical autonomic response characterized by reduced variability, vagal withdrawal, and relative sympathetic predominance. This response is consistent with established HRV physiology: acute hypoxia activates peripheral chemoreceptors and enhances ventilatory drive, accompanied by increased heart rate and reduced baroreflex sensitivity. These changes narrow beat-to-beat oscillatory amplitude and preferentially suppress vagally mediated high-frequency components. When the reduction in HF exceeds that in LF, the LF/HF ratio consequently rises. Previous studies on acute ascent have consistently reported this directional shift (Chen et al., 2008; Hamm et al., 2018; Long et al., 2006a; Sareban et al., 2020; Trimmel et al., 2011; Shi et al., 2020).

SDNN (representing a reduction in overall heart rate variability as a time-domain measure) showed a significant reduction. SDNN reflects the total fluctuation of RR intervals and serves as an integrated marker of multi-timescale sympathetic–vagal interactions and baroreflex modulation. A decline in SDNN following acute ascent indicates a contraction of the “total variability energy,” consistent with hypoxia-induced tachycardia, baroreflex suppression, and reduced vagal activity. For example, in a short-term field study at 2,700 m, SDNN and multiple HRV parameters decreased during a 40-minute recording; the study also reported that diminished overall variability coincided with an increase in subjective alertness (Trimmel et al., 2011). A randomized crossover study in sports medicine conducted under simulated 5,500 m conditions similarly demonstrated a reduction in total power (TP) accompanied by parallel decreases in LF and HF, collectively driving SDNN downward (Wille et al., 2012). Standard guidelines further note that the physiological interpretation of SDNN differs between short-term and 24-h recordings, yet a decrease generally reflects attenuated overall autonomic fluctuation (Task Force of the European Society of Cardiology and the North American Society of Pacing and Electrophysiology, 1996).

RMSSD and pNN50 (representing vagal-dominant time-domain indices) both showed significant reduction. RMSSD and pNN50 primarily reflect cardiac vagal (parasympathetic) activity and the amplitude of respiratory sinus arrhythmia (RSA). After acute high-altitude exposure, the marked decreases in RMSSD and pNN50 indicate a clear “vagal withdrawal” response, characterized by reduced vagal activity and relatively enhanced sympathetic activation. This may result from hypoxia-induced stimulation of the carotid and aortic chemoreceptors, which enhances sympathetic outflow via ascending pathways to the nucleus tractus solitarius in the medulla to maintain blood pressure and oxygen delivery, accompanied by suppression of parasympathetic activity and a notable reduction in short-term HRV (Kim et al., 2018). At the same time, acute hypoxia induces hyperventilation and increases respiratory rate, leading to a reduction in RSA amplitude and weakened heart rate–respiration phase coupling, thereby causing synchronous decreases in HF power and its time-domain counterparts RMSSD and pNN50 (Shaffer and Ginsberg, 2017). In addition, studies have shown that peripheral vasoconstriction and reduced baroreflex sensitivity diminish vagally mediated heart rate inhibitory feedback, further lowering short-term HRV indices (Martinez et al., 2024). Overall, the reductions in RMSSD and pNN50 reflect a short-term shift of autonomic control from “vagal dominance” toward “sympathetic predominance,” representing an adaptive response aimed at maintaining circulatory stability and oxygen delivery efficiency in an acute hypoxic environment.

HF (representing high-frequency components in the frequency domain associated with vagal and respiratory regulation) showed a significant reduction. The reduction in HF in this study reflects a typical pattern of suppressed vagal activity under acute high-altitude exposure. HF primarily reflects respiratory sinus arrhythmia (RSA), and its amplitude is closely linked to vagal modulation of the sinoatrial node. Giorgi and Tedeschi (2025) noted that slow breathing and HRV biofeedback can markedly increase HF, indicating enhanced vagal tone; conversely, in hypoxic environments characterized by reduced oxygen availability and sympathetic activation, a significant decline in HF indicates inhibited vagal output. In addition, the decrease in HF is also related to increased respiratory rate—acute hypoxia induces hyperventilation and accelerates respiratory rhythm, causing the breathing frequency to exceed the upper limit of the HF spectral band (approximately 0.40 Hz). This “frequency shift” reduces the energy of respiratory oscillations within the HF range (Zaccaro et al., 2018). Similar patterns of autonomic imbalance have also been observed in chronic hypoxic conditions, such as chronic obstructive pulmonary disease, where HRV reduction reflects diminished parasympathetic activity and heightened sympathetic activation (Alqahtani et al., 2023). This similarity suggests that hypoxia-induced autonomic dysregulation represents a general physiological mechanism not only present in high-altitude exposure but also applicable to chronic respiratory diseases, indicating that HF and related HRV parameters may serve as sensitive physiological markers of impaired autonomic regulation, useful for assessing disease severity and long-term prognosis. Taken together, the reduction in HF reflects both a neural consequence of vagal withdrawal and a mechanical effect related to changes in respiratory rhythm. In light of these mechanisms, future monitoring of high-altitude exposure should include simultaneous recording of respiratory parameters to distinguish between “true vagal reduction” and the mechanical effects of “respiratory rate–driven HF suppression.”

LF (representing low-frequency components associated with baroreflex modulation and sympathetic–vagal interactions) showed a significant reduction. Following acute ascent, hypoxic stimulation of the carotid and aortic chemoreceptors enhances sympathetic activity to maintain stable blood pressure and oxygen delivery (Mallet et al., 2023). However, despite elevated sympathetic outflow, LF power did not rise accordingly. This is likely because LF is not a “pure sympathetic” marker but reflects rhythmic oscillations jointly modulated by sympathetic and vagal influences via the baroreflex (Goldstein et al., 2011). Under acute hypobaric hypoxia, sympathetic activation accelerates heart rate and increases blood pressure, leading to short-term resetting of the baroreflex and diminished baroreflex-mediated rhythmic modulation of cardiac intervals. At the same time, increased heart rate and reduced overall HRV power cause both LF and HF absolute power to decline (Bourdillon et al., 2023). Therefore, the decrease in LF does not indicate reduced sympathetic activity; rather, it reflects a combined effect of weakened baroreflex modulation, tachycardia, and overall reduced variability.

LF/HF showed a significant increase, representing a “relative effect” reflected by the rise in the ratio. An elevated ratio typically indicates vagal withdrawal and relative sympathetic predominance, serving as an indirect marker of sympathetic activation or stress-related autonomic shifts. However, it is important to note that when the reduction in HF is greater than that in LF, the LF/HF ratio may still increase even if LF decreases in absolute terms, simply because the denominator (HF) declines more rapidly. In addition, the “redistribution” of spectral power caused by increased heart rate and accelerated respiratory frequency can also contribute to a higher ratio. Studies involving rapid ascent to 3,180 m provide a representative example in which normalized LF (LFnu) and LF/HF increase in parallel. At the same time, HFnu decreases—a pattern consistent with the findings of the present analysis. Thus, although LF/HF has long been regarded as a classic marker of sympathovagal balance, recent research suggests that this interpretation may be overly simplified. Lamotte and Goldstein (2022) emphasized that the LF/HF ratio does not directly reflect the absolute balance between sympathetic and parasympathetic activity; rather, it reflects relative shifts in the frequency-domain components of HRV, with more complex underlying physiological mechanisms. Therefore, future research should incorporate multimodal assessments of autonomic regulation—such as direct sympathetic nerve recordings, norepinephrine metabolic markers, or neuroimaging techniques—to more comprehensively characterize sympathetic–parasympathetic dynamics. Given that the present study is based on existing HRV literature, LF/HF was retained as a representative indicator of relative sympathovagal changes to ensure comparability and continuity with previous research.

### 4.2 Effects of acute high-altitude exposure at different elevations on HRV

The altitude subgroup analysis in this study showed that acute high-altitude exposure induces reductions in overall HRV and changes in frequency-domain components across different elevation levels. However, when altitude increases to ≥3,500 m, the decreases in SDNN and the increases in LF/HF become significantly greater, whereas RMSSD, pNN50, HF, and LF, despite showing a numerical trend toward “lower values at higher altitude,” do not exhibit statistically significant differences between subgroups. This finding suggests that the suppression and reorganization of autonomic function induced by acute hypoxia may already be largely established at elevations between 2,500 and 3,500 m. Further ascent to higher altitudes mainly exaggerates the reduction in overall variability (SDNN) and the increase in sympathetic-dominant indices (LF/HF), while additional alterations in vagal-related parameters remain relatively limited.

SDNN showed a significant reduction at higher altitudes and demonstrated a clear altitude-dependent gradient. Similar altitude-related patterns have also been observed in individual studies. Boos et al. (2017) measured HRV repeatedly in the same group of mountaineers at sea level, 3,619 m, 4,600 m, and 5,140 m, and found a progressive decline in SDNN with each increase in elevation. Among long-term residents living at an even higher altitude of 5,300 m, Zhang et al. (2025) reported significant reductions in SDNN as well as multiple time- and frequency-domain indices, indicating further accentuation of cardiac rhythm decomplexification in ultra-high-altitude environments. Integrating these findings with previous reviews on autonomic regulation at altitude, it can be inferred that elevations below 3,500 m primarily induce mild HRV suppression, whereas at ≥3,500 m, sustained chemoreceptor activation, baroreflex resetting, and disturbances in respiratory–sleep rhythms collectively lead to a more pronounced altitude-dependent decline in SDNN and other indices of overall variability.

RMSSD, pNN50, and HF showed no significant differences between the <3,500 m and ≥3,500 m subgroups, indicating that vagal withdrawal may be an “early-onset and early-plateauing” response. Evidence shows that at an altitude of around 2,400 m, RMSSD, pNN50, and HF already decline markedly, reflecting substantial vagal inhibition. Although higher altitudes intensify hypoxia, vagal tone in most healthy individuals is already at a low level, leaving limited room for further reduction; thus, a statistically significant altitude interaction is difficult to detect (Karinen et al., 2012). This suggests that the central autonomic response to hypoxia is highly sensitive in its early phase, with vagal tone dropping rapidly rather than decreasing linearly with rising altitude, and then entering a relatively “stable zone.” From a mechanistic perspective, activation of peripheral chemoreceptors and hypoxia-induced ventilatory responses can rapidly suppress vagal output. At the same time, a generalized elevation of sympathetic baseline activity may further inhibit vagal components through reciprocal autonomic interactions, causing vagal indices to approach their physiological lower limits at moderate altitudes. Therefore, even when altitude increases to ≥3,500 m, vagal parameters continue to show a downward trend, but the magnitude of additional change is limited, reducing the likelihood of a statistically detectable altitude-related differentiation.

LF decreased significantly at both <3,500 m and ≥3,500 m, but the subgroup difference was not statistically significant, and its physiological interpretation requires careful consideration. Traditionally, LF has been closely associated with sympathetic activity; however, increasing evidence suggests that LF actually reflects cardiovascular rhythmic oscillations regulated by the baroreflex and is jointly influenced by both sympathetic and vagal inputs (Goldstein et al., 2011). During acute high-altitude exposure, on the one hand, acute hypoxia can rapidly enhance sympathetic outflow, theoretically driving LF upward (Du et al., 2025); on the other hand, hypoxia can reduce baroreflex sensitivity within minutes to hours, weakening blood pressure–heart rate coupling and thereby lowering LF (Bourdillon et al., 2023). These opposing “upward-driving” and “downward-driving” forces make LF more likely to show an overall reduction in total power during the acute phase. In this study, LF in the ≥3,500 m group decreased to a deeper extent, but the magnitude of decline did not significantly exceed that of the <3,500 m group. A possible explanation is that at the level of acute hypoxia between 2,500 and 3,500 m, baroreflex modulation is already substantially suppressed, causing LF to approach a descending plateau. Alternatively, the inherently short duration of acute exposure—characterized by rapid sympathetic activation but also rapid adjustment of baroreflex function—may place LF in a state of “mixed regulatory limitation” rather than a pattern of linear decline.

LF/HF increased significantly at higher altitudes and displayed a clear altitude-dependent gradient, making it one of the most sensitive indicators of altitude-related modulation in this study. At <3,500 m, LF/HF was already noticeably higher than low-altitude baseline values, and it increased further in the ≥3,500 m group, indicating that with rising altitude, the spectral balance of HRV continues to shift toward the low-frequency domain. This pattern is consistent with enhanced relative sympathetic predominance and the accumulating physiological stress load of high-altitude exposure. Previous hypoxia studies conducted at altitudes around 3,000 m and 4,000 m have similarly reported progressive increases in LF/HF with increasing altitude, typically accompanied by reductions in HF, elevated heart rate, and decreased sleep quality, and have been considered characteristic features of autonomic reconfiguration marked by “sympathetic predominance and vagal suppression” (Zhang et al., 2014). It is important to emphasize that although LF/HF remains widely used as a rough indicator of sympathovagal balance, the ratio more accurately reflects the redistributed relative spectral composition under conditions of reduced total power, rather than a direct quantitative measure of sympathetic tone. Therefore, in this study, the altitude gradient of LF/HF is interpreted as a manifestation of the “passive elevation of low-frequency proportion due to compressed total variability,” particularly because vagal-related indices such as HF, RMSSD, and pNN50 had already decreased markedly at lower altitudes and had limited room for further decline. Together with the significantly reduced SDNN observed at ≥3,500 m, these findings suggest that acute high-altitude exposure places HRV into a state characterized by “restricted variability, low-frequency shifted spectra, and increased sympathetic proportion,” which aligns with physiological mechanisms involving hypoxic load, heightened ventilatory drive, and baroreflex resetting.

### 4.3 Effects of acute high-altitude exposure at different fitness levels on HRV

From the fitness-level subgroup results, this study showed that both healthy adults and trained individuals experienced marked reductions in SDNN, RMSSD, pNN50, and HF following acute high-altitude exposure, with no significant differences between the two groups. Only the changes in LF and LF/HF demonstrated statistically significant modulation across fitness subgroups. This pattern indicates that the overall reduction in HRV and vagal withdrawal induced by acute hypoxia represents a highly “rigid” autonomic response, sufficiently strong to override the resting autonomic differences shaped by long-term training within a short period. The influence of fitness level is therefore primarily reflected in LF and LF/HF, which capture sympathetic–vagal interactions and baroreflex-related modulation.

SDNN and vagal-related indices such as RMSSD, pNN50, and HF decreased markedly in both fitness groups, and the subgroup differences were limited, indicating that vagal inhibition induced by acute high-altitude exposure represents a “low-threshold, early-plateauing” fundamental response. Previous studies have shown that even at altitudes of 2,500–3,000 m, lowland residents exhibit rapid increases in respiratory rate, chemoreceptor excitation, and sympathetic activation, accompanied by significant reductions in HF and RMSSD as well as increases in LF/HF. These patterns reflect rapid vagal withdrawal and an overall reduction in HRV, and further increases in altitude tend to intensify the degree rather than alter the pattern of autonomic responses (Karinen et al., 2012). Systematic reviews and meta-analyses on exercise training and HRV have shown that endurance training can enhance resting vagal activity and HF power at sea level (Amekran and El Hangouche, 2024). However, under acute hypoxic conditions, this advantageous “high vagal reserve” does not fully counteract the acute cardiopulmonary load imposed by high altitude; trained individuals likewise exhibit pronounced vagal inhibition and reductions in SDNN and HF (Tanner et al., 2021). This is consistent with the present finding that “the magnitudes of reduction in SDNN, RMSSD, pNN50, and HF do not differ substantially between fitness subgroups”: the vagal withdrawal triggered by acute high-altitude exposure is driven more by the intensity of hypoxia and chemoreceptor–ventilatory activation than by an individual’s training background.

LF and LF/HF showed subgroup differences across fitness levels, suggesting that training status exerts a modulatory effect on the “sympathetic-dominant components” of autonomic regulation. In this study, LF declined more markedly in healthy adults, whereas the reduction was smaller in trained individuals; correspondingly, the rise in LF/HF was more pronounced among trained participants. This pattern indicates that under acute hypoxia, trained individuals may exhibit more prominent sympathetic excitation or retain stronger low-frequency rhythmic oscillations on top of vagal withdrawal, resulting in a greater increase in LF/HF. By contrast, healthy adults tend to display a more “global” reduction in power. Previous studies in high-altitude exercise physiology have noted that endurance-trained individuals possess higher resting vagal tone and greater baroreflex sensitivity during short-term altitude adaptation, but during combined hypoxia and physical stress, their sympathetic activation can be even more pronounced. Their blood pressure and heart rate responses may exceed those of untrained individuals, aligning with observations that endurance athletes are not necessarily protected from acute mountain sickness upon initial ascent and may even exhibit slightly elevated risk (Sareban et al., 2020). Mechanistically, on the one hand, long-term training reshapes cardiovascular autonomic regulation, giving athletes heightened sensitivity to blood pressure fluctuations and more pronounced LF oscillations at rest. On the other hand, hypoxia-induced reductions in baroreflex sensitivity combined with enhanced chemoreceptor drive may manifest in trained individuals as “stronger sympathetic outflow” and “relatively preserved LF rhythmicity,” ultimately producing a more marked elevation of LF/HF in the frequency domain (Alvarez-Araos et al., 2024).

### 4.4 HRV characteristics during acute high-altitude exposure and implications for intervention strategies

Synthesizing the findings from the systematic review and meta-analysis, the HRV alterations induced by acute high-altitude exposure exhibit a highly consistent autonomic pattern in which SDNN, RMSSD, pNN50, HF, and LF all decrease, while LF/HF increases significantly. This pattern reflects a characteristic physiological profile of “overall variability constriction—vagal withdrawal—relative sympathetic predominance.” Such a profile indicates that under acute hypoxic load, autonomic redundancy, short-term heart rate regulatory capacity, and cardiopulmonary coupling rhythms are substantially constrained, thereby imposing considerable physiological stress on circulatory homeostasis and respiratory regulation. Based on these physiological features, several regulatory implications can be summarized as follows.

1. The reduction in SDNN indicates weakened overall autonomic regulatory capacity. Therefore, during the initial phase of high-altitude exposure, priority should be given to controlling total physiological load, including “progressive ascent, prolonged adaptation at moderate altitudes, adequate hydration, and avoidance of large fluctuations in exercise intensity,” in order to mitigate further compression of heart rate and blood pressure oscillations and maintain relative stability in total HRV power.
2. The simultaneous decreases in RMSSD, pNN50, and HF reflect significant suppression of vagal drive and reduced short-term heart rate variability. Combined with the rise in LF/HF, which suggests relative sympathetic predominance, interventions such as “slow-paced breathing training, HRV biofeedback, low-to-moderate intensity aerobic exercise, and relaxation training” may be beneficial before and after exposure to facilitate vagal recovery (Giorgi and Tedeschi, 2025), improve respiratory sinus arrhythmia, and reduce sustained sympathetic overactivation.
3. The reduction in HF arises not only from vagal withdrawal but also from increased respiratory rate and hyperventilation induced by acute hypoxia. Thus, stabilizing respiratory rhythm and sleep structure is equally essential. During the early stage of exposure, individuals should “avoid hyperventilation caused by high-intensity exercise,” maintain a stable breathing frequency through paced respiration (Sevoz-Couche and Laborde, 2022), and “adhere to regular sleep schedules” to promote recovery of baroreflex sensitivity and cardiopulmonary coupling rhythms.
4. The altitude and fitness subgroup findings further highlight differentiated intervention directions. Individuals at ≥3,500 m exhibit more pronounced SDNN reductions and LF/HF elevations, suggesting that autonomic reserve becomes more fragile at higher altitudes. Accordingly, adaptation time between 2,500 and 3,500 m should be extended before crossing the 3,500 m threshold, with strengthened dynamic monitoring of heart rate, SpO_2_, and HRV. Although trained individuals have greater autonomic reserve at sea level, their greater LF/HF elevation under acute hypoxia indicates that “high fitness” does not equate to “low risk,” and conservative ascent pacing with adequate recovery remains necessary. In individuals with average fitness levels, the more pronounced reductions in LF and total power suggest the need for even more cautious ascent speed to avoid prematurely entering high-load altitude zones (Alvarez-Araos et al., 2024).

In summary, the HRV alterations observed during acute high-altitude exposure highlight three key vulnerabilities of the autonomic nervous system under hypoxia: reduced overall variability, suppressed vagal activity, and relative sympathetic predominance. Therefore, strategies ranging from pre-ascent autonomic conditioning, to load control during ascent, to post-ascent recovery should all be directed toward preserving autonomic balance. Moreover, future studies may consider integrating HRV with functional measures such as balance performance to more comprehensively evaluate adaptive capacity under acute high-altitude exposure. Emerging evidence indicates that HRV is associated with balance tasks such as single-leg stance (Tedeschi et al., 2024), suggesting that the two may reflect shared aspects of physiological homeostasis. These approaches not only help slow the unfavorable trajectory of HRV changes but may also reduce the risk of acute mountain sickness to some extent.

## 5 Conclusion

This study found that acute high-altitude exposure markedly reduced SDNN, RMSSD, pNN50, HF, and LF, while increasing LF/HF, presenting an autonomic profile characterized by “reduced variability, vagal withdrawal, and relative sympathetic predominance.” These changes were more pronounced at altitudes ≥3,500 m, suggesting that elevation serves as an important amplifying factor of autonomic load. Fitness subgroup analysis showed that both trained and healthy adults experienced vagal suppression under acute hypoxia; however, trained exhibited better preservation of low-frequency rhythmicity and stronger overall sympathetic regulatory capacity, indicating certain adaptive advantages, albeit with potentially heightened sympathetic responsiveness. Based on these findings, strategies such as gradual ascent, respiratory rhythm stabilization, and recovery-focused training before and after entering high-altitude environments may help mitigate autonomic disturbances and improve early adaptation. Future studies with larger samples and more comprehensive HRV measures are needed to further validate and extend these conclusions.

## 6 Limitations

Although this study used a systematic review and meta-analysis to demonstrate the significant effects of acute high-altitude exposure on HRV, several limitations remain. (1) Limited sample coverage. Despite including multiple studies, the overall number of eligible publications was still relatively small, and several studies had modest sample sizes, making it difficult to capture individual differences across sex, age, and fitness groups. The limited data available for women, older adults, and individuals with lower fitness, as well as the lack of established clinical reference values for HRV indices, restricted the feasibility of more detailed subgroup analyses and reduced the generalizability of the findings. (2) Residual confounding cannot be fully excluded. There were methodological inconsistencies across studies, including differences in recording duration, body position, resting period before measurement, device type, and control of confounding factors. Although most studies restricted exercise, caffeine intake, and other influences before testing, a few did not report such control measures or used 24-h recordings, which may be affected by daily physical activity. Additionally, environmental factors such as temperature, humidity, and sleep quality were insufficiently documented in some studies, leaving uncertainty regarding their impact on HRV responses. (3) Potential publication bias. Although the publication bias assessments indicated an overall low risk, the small number of included studies (<20) means that we cannot completely rule out the possibility that some positive findings may be overestimated due to publication bias. Future studies should expand sample sizes and increase the number of eligible studies to improve the reliability of publication bias evaluation. (4) Limited coverage of HRV metrics. Most studies reported only conventional time-domain and frequency-domain indices (e.g., SDNN, RMSSD, LF/HF), while evidence for nonlinear indices (e.g., SD1, SD2) remained insufficient. Future research should expand the range of HRV parameters to obtain a more comprehensive understanding.

## Data Availability

The original contributions presented in the study are included in the article/Supplementary Table S2, further inquiries can be directed to the corresponding authors.

## Abbreviations

HRV, Heart Rate Variability; SDNN, Standard Deviation of Normal-to-Normal intervals; RMSSD, Root Mean Square of Successive Differences; pNN50, Percentage of successive NN intervals differing by more than 50 ms; HF, High Frequency power; LF, Low Frequency power; LF/HF, Low Frequency to High Frequency ratio.
