# Supplementary material for: Effects of acute high-altitude exposure on heart rate variability: a systematic review and meta-analysis
Source: Front Physiol. 2026 Jan 5;16:1696346. doi: 10.3389/fphys.2025.1696346 (PMC12812737; doi:10.3389/fphys.2025.1696346)
Supplement: Supplementary file 1 [file Table1.docx]

# Appendix A

# Database Search Strategies

In accordance with the PRISMA guidelines, a systematic search of literature related to acute high-altitude exposure was conducted. The databases included PubMed, Web of Science, Cochrane Library, Embase, China National Knowledge Infrastructure (CNKI) and Wanfang. The search period covered all records from database inception to June 2025. The search focused on studies comparing baseline (sea-level or low-altitude) measurements with post-exposure outcomes after entering high-altitude environments. Eligible participants were adults originating from low-altitude regions who had heart rate variability (HRV) measurements both before and after acute exposure to high altitude (typically within 7 days of ascent). A combination of subject headings and free-text terms was used. The search process is summarized in Table A1.

**Table A1. Database Search Strategies**

| Database | Search Method | Search Strategy (Keywords / Subject Terms) |
| --- | --- | --- |
| PubMeD | MeSH terms +  free-text terms | ("Altitude"[MeSH] OR "Hypoxia"[MeSH] OR "High altitude" OR "Plateau" OR "Hypobaric hypoxia" OR "Acute mountain sickness") AND ("Heart Rate Variability"[MeSH] OR "HRV" OR "Heart rate variability" OR "Cardiac autonomic function" OR "Autonomic nervous system") AND ("Acute"[All Fields] OR "Short-term"[All Fields] OR "Immediate"[All Fields]) |
| Web of Science | Topic terms +  free-text terms | (("High altitude" OR "Plateau" OR "Hypoxia" OR "Hypobaric hypoxia" OR "Acute mountain sickness" ) AND ("Heart rate variability" OR HRV OR "Cardiac autonomic function" OR "Autonomic nervous system") AND ("Acute" OR "Short-term" OR "Immediate")) |
| Cochrane Library | MeSH terms + free-text terms | (MeSH descriptor: [Altitude] explode all trees OR MeSH descriptor: [Hypoxia] explode all trees OR “high altitude”:ti,ab,kw OR plateau:ti,ab,kw OR “hypobaric hypoxia”:ti,ab,kw OR “acute mountain sickness”:ti,ab,kw) AND (MeSH descriptor: [Heart Rate Variability] explode all trees OR HRV:ti,ab,kw OR “heart rate variability”:ti,ab,kw OR “cardiac autonomic function”:ti,ab,kw OR “autonomic nervous system”:ti,ab,kw) AND (acute:ti,ab,kw OR short-term:ti,ab,kw OR immediate:ti,ab,kw) |
| Embase | Emtree terms + free-text terms | (‘altitude’/exp OR ‘hypoxia’/exp OR ‘high altitude’:ti,ab,kw OR plateau:ti,ab,kw OR ‘hypobaric hypoxia’:ti,ab,kw OR ‘acute mountain sickness’:ti,ab,kw) AND (‘heart rate variability’/exp OR HRV:ti,ab,kw OR ‘heart rate variability’:ti,ab,kw OR ‘cardiac autonomic function’:ti,ab,kw OR ‘autonomic nervous system’:ti,ab,kw) AND (acute:ti,ab,kw OR ‘short term’:ti,ab,kw OR immediate:ti,ab,kw) |
| CNKI | Subject terms +  free-text terms | ("High altitude" OR "Acute ascent to high altitude") AND ("Autonomic nervous system" OR "Heart rate variability" OR HRV) |
| Wanfang | Subject terms +  free-text terms | ("High altitude" OR "Acute ascent to high altitude") AND ("Autonomic nervous system" OR "Heart rate variability" OR HRV) |
